# The Efficacy of Safinamide in the Management of Parkinson's Disease: A Systematic Review

**DOI:** 10.7759/cureus.29118

**Published:** 2022-09-13

**Authors:** Joudi Sharaf, Kerry-Ann D Williams, Maha Tariq, Maitri V Acharekar, Sara E Guerrero Saldivia, Sumedha Unnikrishnan, Yeny Y Chavarria, Adebisi O Akindele, Ana P Jalkh, Aziza K Eastmond, Chaitra Shetty, Syed Muhammad Hannan A Rizvi, Lubna Mohammed

**Affiliations:** 1 Neurology, California Institute of Behavioral Neurosciences & Psychology, Fairfield, USA; 2 Anesthesiology, California Institute of Behavioral Neurosciences & Psychology, Fairfield, USA; 3 Family Medicine, California Institute of Behavioral Neurosciences & Psychology, Fairfield, USA; 4 Gastroenterology, California Institute of Behavioral Neurosciences & Psychology, Fairfield, USA; 5 Ophthalmology, California Institute of Behavioral Neurosciences & Psychology, Fairfield, USA; 6 Internal Medicine, California Institute of Behavioral Neurosciences & Psychology, Fairfield, USA; 7 Research, California Institute of Behavioral Neurosciences & Psychology, Fairfield, USA; 8 Family Medicine/ Dermatology, California Institute of Behavioral Neurosciences & Psychology, Fairfield, USA; 9 Medicine and Surgery, California Institute of Behavioral Neurosciences & Psychology, Fairfield, USA

**Keywords:** parkinson's disease non-motor symptoms, motor fluctuations, parkinson's disease treatment, safinamide, parkinson’s disease (pd)

## Abstract

Parkinson's disease (PD) is a chronic neurodegenerative disease that is challenging to treat due to its progressive nature and its weaning response to therapy. Safinamide, a monoamine oxidase type-B inhibitor (MAOB-I), has shown promise in managing dyskinesias caused by levodopa (L-dopa), carbidopa, and PD features such as pain and depression. This systematic review aimed to evaluate safinamide's efficacy as a monotherapy and an add-on in tackling these issues. We composed this systematic review according to the Preferred Reporting Items for Systematic Reviews and Meta-Analysis (PRISMA) guidelines. Our group searched the following databases: Manchester University Library, ScienceDirect, Google Scholar, PubMed, PubMed Central, and MedLine for articles produced in the last ten years using various search terms and criteria, which we outlined in the search strategy and eligibility criteria sections. We excluded 722 out of the initially screened 730 records for multiple reasons, such as titles and abstracts being irrelevant to the topic, articles without free full access, articles originally not in the English language, and articles that did not score 70% or above on their respective quality assessment tools. The studies explored supported safinamide's use in managing motor fluctuations, pain, depression, and improving patients' quality of life.

## Introduction and background

Parkinson's disease (PD) is a progressive disorder characterized by neurodegeneration of the dopaminergic neurons in the substantia nigra [1]. According to the US Census Bureau population projections, it is estimated that the number of diseased individuals is expected to rise to 1,238,000 by 2030 [2]. The clinical features of PD can be categorized into two subtypes: motor and non-motor. The motor features have two main themes, those caused by loss of dopaminergic activity, such as bradykinesia and cogwheel rigidity, and those caused by an increase in cholinergic activity, such as a resting "pill-rolling" tremor [1,3].

Non-motor symptoms mainly include cognitive and neuropsychiatric features such as depression, inattentiveness, hallucinations, and pain [3-5]. Patients with PD suffer from various motor and non-motor symptoms, which tend to worsen over time and lose responsiveness to therapeutic modalities [1,6]. In addition, PD is a chronic, progressive disease and patients usually follow a declining trajectory whereby the symptoms worsen over time and become less responsive to interventions. These features make the management of PD a complex challenge for healthcare professionals and markedly a burden on the patient's quality of life.

As mentioned, the pathophysiology of PD involves the loss of dopaminergic neurons in the substantia nigra; hence, the goal of the mainstay treatment options is to raise dopamine levels at the receptors where neuronal loss has occurred [7,8]. The main agents used to achieve this goal are levodopa (L-DOPA) and carbidopa. L-DOPA is a precursor of dopamine, making it helpful in replenishing dopamine in the central nervous system (CNS), which is deficient in PD patients. Unfortunately, it is also metabolized to dopamine peripherally, which causes unwanted side effects. With that in mind, combining L-DOPA with carbidopa aims to inhibit L-DOPA's conversion to dopamine peripherally to curb unwanted side effects and increase L-DOPA levels reaching the CNS [7]. However, this combination's efficacy weans over time, and its long-term use can lead to the emergence of dyskinesias and other complications [9]. These adverse effects can be partially mitigated with monoamine oxidase-type B inhibitor (MAOB-I) and glutamate N-methyl-D-aspartate receptor antagonists [10]

Safinamide is a potent and reversible MAOB-I that can be used to counteract these dyskinesias. It modulates Na+ and Ca2+ channels, reduces glutamate release, and inhibits monoamine oxidase-type B contributing to its use in dyskinesias [7,9]. Safinamide's benefits in treating PD extend beyond treating L-DOPA/carbidopa-induced dyskinesias; it was also approved by the FDA in 2017 as an add-on therapy to L-DOPA/carbidopa when treating "off" symptoms [11]. A systematic review with meta-analysis on pain management in PD found that safinamide was the most effective among the studied medications in decreasing pain in PD patients [5]. In 2021, a pilot study found that safinamide can also improve rapid eye movement sleep behavior disorder in PD patients [12].

In this systematic review, we aim to evaluate the available evidence that discusses the efficacy of safinamide as a monotherapy and as an add-on drug in patients with PD.

Methods

We performed this systematic review in compliance with Preferred Reporting Items for Systematic Reviews and Meta-Analysis (PRISMA) guidelines [13]. 

Eligibility Criteria

We included studies with the following characteristics: randomized control trials, systematic reviews, and meta-analyses published in the years 2012-2022, full-text papers, studies in the English language, and studies done on humans.

Search Strategy

We used the following databases: Manchester University Library, ScienceDirect, Google Scholar, PubMed, PubMed Central, and Medline. The search terms used in Manchester University Library, ScienceDirect, and Google Scholar are "Safinamide" and "Parkinson's disease". Our MeSH strategy used in PubMed, PubMed Central, and Medline is Parkinson disease OR ("Parkinson disease/drug therapy" [Majr] AND safinamide OR "monoamine oxidase inhibitors/pharmacology" [Majr] OR "alanine/pharmacology" [Majr] OR "benzylamines/pharmacology" [Majr] Table 1 outlines the search strategy in detail.

**Table 1:** Details of the search strategies MESH - Medical Subject Headings

Database	Search terms	Filters	Results
PubMed, PubMed Central, Medline	"Safinamide" and "Parkinson’s disease"; MESH strategy was: Parkinson disease OR ("Parkinson disease/drug therapy" [Majr] AND safinamide OR "monoamine oxidase inhibitors/pharmacology" [Majr] OR "alanine/pharmacology" [Majr] OR "benzylamines/pharmacology" [Majr]	2012-2022 (10 years), English, humans, full-text, clinical trial, meta-analysis, randomized control trial, systematic review, case report	200
Google Scholar	Safinamide, Parkinson's disease	2012-2022	2020
Sciencedirect	Safinamide, Parkinson's disease	Last 10 years since 2012, review articles, research articles, case reports, mini-reviews, practice guidelines, and others	229
Manchester University Library	Safinamide, Parkinson's disease	2012-2022, English	992

Quality Assessment

Table 2 showcases the quality assessment tools used to ascertain which articles were to be included as part of the final eight studies.

**Table 2:** Quality assessment tools, their passing score, and which studies passed PRISMA - Preferred Reporting Items for Systematic Reviews and Meta-Analyses

Quality assessment tool	Study design	The maximum score that can be attained	Score accepted (more than 70%)	Studies with passing scores
PRISMA [13]	Systematic reviews and meta-analyses	27	19	Giossi et al. [1]; Huang et al. [4]; Qureshi A.R et al. [5]; Ahmed et al. [8]; Binde et al. [14]
Cochrane risk of bias tool [15]	Randomized control trials	7	5	Hattori et al. [7]; Borgohain et al. [9]; Schapira et al. [10]

Study Selection

Two authors retrieved citations and screened the reports independently. The two authors discussed the articles' compatibility with the inclusion and exclusion criteria. The studies meeting the inclusion criteria were incorporated for qualitative assessment. Two authors separately drew out information and the results of each study. All studies that met the quality assessment tools were included in this systematic review. We attempted to contact authors for publications with unavailable data; however, we received no response. We produced our manuscript according to the PRISMA statement.

Data Extraction and Data Items

Two authors independently extracted data and resolved any disagreements via discussion. As a result, we found the following data: publication date, duration of the study, age and gender of patients, inclusion and exclusion criteria, the language of the original study, number of patients exposed and unexposed to the treatment of interest, and results of outcomes of interest.

Assessment of Risk of Bias

Two authors independently assessed the quality of the randomized control trial by using the Cochrane risk of bias tool (RoB2) across five domains: selection bias, performance bias, detection bias, attrition bias, reporting bias, and any other source of bias that might have influenced the study data. We excluded studies showing a high risk of bias in two or more domains from the review. The risk of bias in included studies is assessed at the study level using the Cochrane risk of bias tool. Discussion with colleagues resolved any disagreement.

Results

**Figure 1:**
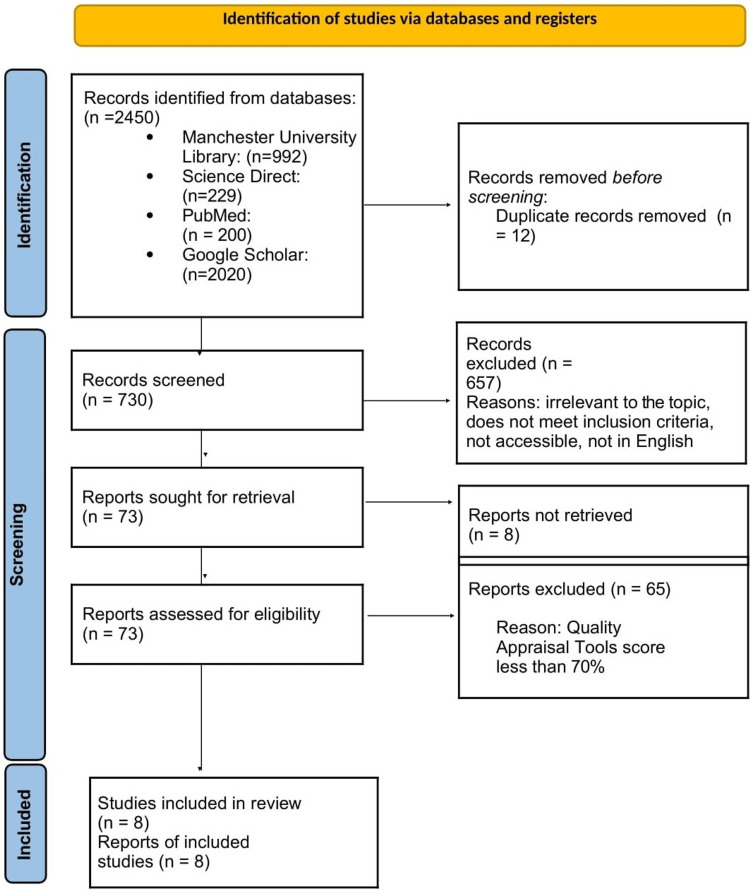
PRISMA 2020 flow diagram for new systematic reviews depicting the study selection process PRISMA - Preferred Reporting Items for Systematic Reviews and Meta-Analyses

After screening reports, we excluded 657 for the following reasons: not in the English language, no free full access, title irrelevant to the topic, abstract not focusing on the point of interest. After the manual screening, 73 reports were sought out and read, of which we excluded 65 after assessing for eligibility using quality checklist tools. They had to meet 70% of the checklist to be qualified to be included in the systematic review. The PRISMA 2020 flow diagram for new systematic reviews summarizes the findings in Figure 1. 

The eight main studies reviewed had varying characteristics and assessed safinamide either as monotherapy or as an add-on. These findings are summarized in Table 3.

**Table 3:** Characteristics and conclusions of the eight studies included in this systematic review PD - Parkinson's disease; L-DOPA - levodopa; MAOB-I - monoamine oxidase type-B inhibitor

Author	Year	Study design	Modality of treatment/factors investigated	Conclusion
Giossi et al. [1]	2021	Systematic review and meta-analysis	Safinamide	Safinamide was found efficacious in PD patients with motor fluctuations
Ahmad et al. [8]	2019	Systematic review and meta-analysis	Safinamide	Provided class 1 evidence regarding the role of safinamide as an adjunct medication in patients with PD with motor fluctuations
Borgohain et al. [9]	2013	Randomized clinical trial	Safinamide as an add-on to L-DOPA in PD patients with motor fluctuations	The drug increased total "on time," decreased "off-time," and improved parkinsonism
Binde et al. [14]	2020	Meta-analysis	Dopamine agonists and MAOB-Is	This study found that safinamide was not effective in comparison to placebo. Selegiline was found to be most effective as an add-on to L-DOPA
Schapira et al. [10]	2017	Randomized clinical trial	Safinamide as an add on to L-DOPA	This study concluded that the drug in combination with L-DOPA improves the studied outcomes: off time, on time without dyskinesia
Hattori et al. [7]	2020	Randomized control trial	Safinamide as a L-DOPA adjunct	The conclusions of this study found safinamide to be safe and increase "on time," and alleviate symptoms in PD patients with wearing-off
Huang et al. [4]	2021	Meta-analysis	MAOB-Is	This study found that MAOB-Is decrease the severity of depressive episodes in PD patients
Qureshi et al. [5]	2018	Systematic review and meta-analysis	Therapies for pain	From the therapies studied in this research, safinamide was found to be most effective in decreasing pain in PD patients

## Review

As previously discussed, PD can cause debilitating symptoms that are often difficult to treat. Not only that, but various treatments for this disease have adverse effects which can be challenging to manage. The following section will evaluate the efficacy of safinamide in managing different PD symptoms and adverse effects caused by its medications.

Effect on motor fluctuations

According to the early detection of wearing off in Parkinson's disease (DEEP) study, motor fluctuations have been reported as one of the main adverse effects of PD medications such as L-DOPA [16]. This adverse effect has not yet been managed adequately in PD patients, although safinamide, an MAOB-I, has shown some promise [17]. Motor fluctuations also increase the yearly cost of treating PD by double or triple the cost of those who do not suffer from this adverse effect [18]. Motor fluctuations are thought to be due to alterations in dopamine, glutamate, and other neurotransmitters. Safinamide's ability to affect dopamine and glutamine makes it a potential solution for managing motor fluctuations [3]. 

A meta-analysis explored the effect of safinamide as an add-on to manage these fluctuations and concluded that the drug is efficacious and well endured by patients [8]. The effect of this drug was based on three quantifying parameters: "on-time without troublesome dyskinesia", "off-time," and the Unified Parkinson's Disease Rating Scale part three (UPDRS III) score [8]. The UPDRS III aims to evaluate how motor symptoms affect PD patients and comprises four components [8]. A dose of 100 mg per day was one that notably improved the three parameters and had an effect comparable to rasagiline's effect as an add-on to L-DOPA when it came to increasing the on-time without troublesome dyskinesia [1].

Two other meta-analyses compared rasagiline and safinamide's efficacy in decreasing off-time and increasing the UPDRS III score. The results were similar, with the slight edge going to rasagiline [1,19]. A randomized trial conducted in 2013 further illustrated safinamide's beneficial effect, as an add-on to dopamine agonists, in increasing on-time without troublesome dyskinesia, quoting 50 and 100 mg/day as the two doses responsible for this effect [9]. A multiple treatment comparison meta-analysis found that safinamide was ineffective compared to placebo, although possible weakness in clinical heterogeneity of the studies included limited the quality of the results [14]. Another randomized controlled trial conducted in 2017 supplemented the findings of the one done in 2013 by stating that the 100 mg per day dosing regimen increased the on-time without troublesome dyskinesia by almost an hour versus placebo. The study states that the effect sets in within a fortnight and was still maintained after 18 months [10]. Among the constituents of the Unified Parkinson's Disease Rating Scale score, safinamide's effect as an add-on to L-DOPA was most significant in improving tremors, postural instability, and gait, which are symptoms that L-DOPA usually has an unpredictable effect on. Safinamide also supplements L-DOPA's well-defined effect on bradykinesia and rigidity [6]. The majority of the data points towards improvements in motor fluctuations. More recently, a randomized clinical trial conducted in 2020 has touted safinamide as a favorable add-on treatment with a mostly tolerated adverse effect profile [7].

The dyskinesia rating scale (DRS) is a descriptive assessment tool used to evaluate the severity of motor side effects resulting from PD dopaminergic drugs [8]. Safinamide was not shown to improve the DRS score over placebo at the 100 mg per day or the 50 mg per day dosing regimens, and it was only evaluated in PD patients with motor fluctuations [1]. The problem with the DRS is that it is not very sensitive to dyskinetic changes hence more studies are required that incorporate the use of more accurate assessment tools [1].

Effect on quality of life

Safinamide was also evaluated for its ability to improve different aspects of PD patients' quality of life. The measures of improvement included the mini-mental state examination (MMSE), the Parkinson's disease questionnaire (PDQ-39), and the Unified Parkinson's Disease Rating Scale part two (UPDRS II). MMSE is a widely used 30-element tool to assess the patient's cognitive performance, and in PD, it is used to monitor the patient's response to treatment and disease progression [20]. UPDRS II is a measurement tool widely used for the clinical monitoring of PD patients. It assesses the patient's ability to perform daily life activities such as speaking, clothing oneself, and deglutition [21]. PDQ-39 is a comprehensive assessment tool to evaluate their attention, short-term memory, depressive symptoms, functional mobility, and social support and interactions [22]. Safinamide was superior to placebo in improving scores of all three assessment tools mentioned above, according to a meta-analysis performed by Ahmed et al. [8].

A systematic review and meta-analysis performed by Giossi et al. further assessed safinamide's efficacy in improving the UPDRS II in patients with PD with and without motor fluctuations. In patients with PD with motor fluctuations, the score was notably increased with the administration of the 100 and 50 mg daily doses. On the other hand, patients with PD without motor fluctuations experienced a much smaller yet clinically relevant increase at the 100 mg dose, although the level of evidence was low [1]. A randomized controlled trial performed by Borgohain et al. provided evidence that the 100 mg per day dosing regimen is superior to the 50 mg per day dosing regimen and placebo in increasing the UPDRS II score [9].

The PDQ-39 score was increased by the 100 mg per day dosing regimen according to Giossi et al. However, they could only analyze patients with PD with motor fluctuations and reported that the evidence level was low due to high bias risk and inaccuracy [1]. Borgohain et al. also reported increases in the PDQ-39 score and increases in other quality of life measures such as emotional welfare, communication, and physical comfort with the 100 mg per day dosing regimen over placebo [9].

Effect on depression

Depression is a significant nonmotor feature of PD as it can be the first sign of the disease appearing as a prodrome to motor symptoms and its negative implications on a patient's daily life [4]. Several contributors lead to the development of this symptom. Firstly, the dopamine deficiency that characterizes PD is one of the main contributors. Secondly, other elements such as esteem issues due to compromised motor function, loss of sleep, and anxious mood changes resulting from the diagnosis also contribute [4]. The initial approach to treating this disorder is to outline the patient's overall state through an interdisciplinary assessment; afterward, tackling motor symptoms and picking an optimal dose of dopaminergic medications takes precedence over dispensing drugs for depression [23]. MOAB-Is' ability to target both motor and depressive symptoms makes them an attractive option for this treatment goal [4]. Safinamide also has the added mechanism of action of modulating glutamate, which has also contributed to ameliorating depressive symptoms in theory [4]. However, in the clinical setting, safinamide's effect on mood alterations and depression seems to be inconsistent [10].

A post hoc analysis conducted in 2017 concluded that when used long-term (two years), safinamide is indeed effective in decreasing depressive episodes; however, these findings were not supported in a meta-analysis assessing the effect of MOAB-Is as a treatment for depression in PD [4,24]. On the contrary, this meta-analysis found that, as a class, MOAB-Is are more effective as a short-term management option for depression in those with an early presentation of PD [4]. Another systematic review and meta-analysis evaluated safinamide's effect specifically and revealed that the drug is superior to placebo in terms of improving the Hamilton Depression Rating Scale (HAM-D), which is a 21-unit checklist used in the evaluation of depressive features by pharmaceutical trials in the clinical setting [8,25]. This finding was supported by a randomized controlled trial conducted in 2013 in which safinamide was better than placebo at increasing the HAM-D score across 24 weeks [9].

Effect on pain

Pain is another symptom that is extremely common in patients with PD, with rates ranging from 68-95%, according to multiple studies [26-29]. Even with the high proportion of PD patients suffering from pain, a quarter to half of them remain untreated, indicating the presence of a gap that needs to be filled [26-27, 29]. This symptom can aggravate mood symptoms (depression and anxiety), further contributing to the morbidity associated with PD [30]. As previously highlighted, safinamide has a dual effect on dopaminergic and glutaminergic pathways, the latter of which has been implicated in the drug's analgesic effects in PD [31]. A systematic review and meta-analysis evaluating analgesic therapies for pain in PD assessed the effect of multiple options such as safinamide, cannabinoids, catechol-o-methyl-transferase (COMT) inhibitors, dopaminergic agonists, opioids, Chinese and electrical therapies, surgery, pardoprunox as well as an interdisciplinary approach in order to ascertain the most superior one [5]. The results point toward safinamide being the superior treatment option [5]. This result makes it an even more attractive adjunctive option as it has the benefit of treating pain and other parkinsonian symptoms.

Limitations

This study had several limitations. First, some of the studies included had several patients lost to follow-up, causing attrition bias. In addition, some studies had sample sizes that were not large enough, had inadequate blinding, and used unverified scales to assess outcomes, all of which have impacted the quality of evidence. Another notable limitation is that not all studies compared safinamide to a different drug; thus, the response to safinamide in outcome measures cannot be conclusive. Therefore, more studies comparing safinamide with other therapeutic options are needed. Finally, as with many studies, various patient demographics and follow-up times can bring heterogeneous results.

## Conclusions

Due to PD's progressive nature, the decrease in the effectiveness of its therapeutic interventions over time, and the considerable side effects of its treatments, the management of this disease remains a challenge. This study reviews the evidence for the efficacy and safety of safinamide, which has shown benefits in treating motor and non-motor symptoms and managing some of the treatments' side effects. Despite the heterogeneity in the studies, safinamide shows efficacy in managing motor fluctuations, as illustrated by improvement in clinically relevant parameters as monotherapy or as an add-on therapy. Furthermore, safinamide can be considered when managing non-motor symptoms such as pain and depression, as this review shows it to be effective in managing these symptoms. Enhanced patient-centered care can improve efficacy and safety outcomes to mitigate or minimize adverse effects, drug-drug interactions, and polypharmacy in patients living with PD.

Overall, studies with a higher number of participants and more robust evidence evaluating this intervention may provide clinicians with further guidance regarding the role of safinamide in managing the various symptom domains of this condition. Future researchers can achieve this need by examining different doses as part of randomized controlled trials to confirm the efficacy and safety of this novel drug. This study summarizes the current evidence and highlights what gaps future research can fill to provide a higher level of evidence on this drug's multifaceted use in PD.
